# Combined Lung Immune Prognostic Index (LIPI)-Glasgow Prognostic Score (GPS) as a Prognostic Tool in Extensive-Stage Small-Cell Lung Cancer Treated with First-Line Chemo-Immunotherapy

**DOI:** 10.3390/ph19040587

**Published:** 2026-04-07

**Authors:** Maral Martin Mıldanoğlu, Fatih Kemik, Melisa Eryaşar, Hakan Özçelik, Erdem Sünger, Mehmet Haluk Yücel, Ebru Engin Delipoyraz, Sena Fidan, Harun Muğlu, Burçin Çakan Demirel, Jamshid Hamdard, Yasin Kutlu, Özgür Açıkgöz, Fatih Selcukbiricik, Mesut Şeker, Ahmet Bilici

**Affiliations:** 1 Department of Medical Oncology, Faculty of Medicine, Medipol University, Istanbul 34083, Turkey; melisaery2002@gmail.com (M.E.); hknozcelikk@gmail.com (H.Ö.); erdemsunger@gmail.com (E.S.); hm1635@hotmail.com (H.M.);; 2 Department of Medical Oncology, Faculty of Medicine, Koc University, Istanbul 34460, Turkey; kemikfatih41@gmail.com (F.K.);

**Keywords:** ES-SCLC, LIPI, GPS, prognostic, immunotherapy

## Abstract

**Introduction:** Inflammatory and immune-based prognostic markers such as the Lung Immune Prognostic Index (LIPI) and the Glasgow Prognostic Score (GPS) have gained increasing attention in ES-SCLC, particularly in patients receiving first-line chemoimmunotherapy. However, no prior study has explored a broader, integrated inflammatory framework that evaluates these parameters collectively. **Methods:** We retrospectively evaluated 166 patients with ES-SCLC treated with first-line platinum–etoposide plus atezolizumab or durvalumab between 2019 and 2025. LIPI could be calculated in 123 patients based on available dNLR and LDH values, while GPS and the Combined Inflammatory Prognostic Score (CIPS) could be assessed in 120 patients with accessible CRP and albumin data. **Results:** Median PFS and OS were 8.16 and 15.96 months, respectively. In univariate analyses, poor ECOG PS, liver and bone metastases, poor LIPI, poor GPS, and high-risk CIPS were associated with shorter PFS and OS. In multivariate analysis, only LIPI and GPS remained independent predictors of both PFS and OS, while ECOG PS was independently associated with OS. Although CIPS demonstrated clear prognostic separation in univariate analysis, it did not retain independent significance, likely due to sample size limitations and overlap with LIPI and GPS components. **Conclusions:** LIPI and GPS are strong independent prognostic markers in ES-SCLC receiving chemoimmunotherapy. While CIPS did not demonstrate independent prognostic value in multivariate analysis, its simplicity, balanced two-tier design, and use of routinely available biomarkers highlight its potential clinical utility. To our knowledge, this is the first study to assess a combined inflammatory prognostic model in this population. Prospective multicenter validation is warranted.

## 1. Introduction

Lung cancer was the leading cancer worldwide in terms of both incidence and mortality in 2022. Although its incidence appears to be shifting toward the second rank in recent years, it still remains the leading cause of cancer-related mortality globally [1,2]. Small-cell lung cancer (SCLC) accounts for approximately 15% of all lung cancer cases, with around 150,000 new diagnoses reported globally each year [3]. SCLC is a highly aggressive malignancy marked by rapid tumor growth and an early tendency for widespread metastasis [4]. Nearly 70% of patients are diagnosed with extensive-stage disease (ES-SCLC) at presentation [5]. For several decades, platinum-based chemotherapy combinations represented the cornerstone of first-line treatment for ES-SCLC prior to the 2000s [6]. More recently, the phase III IMpower133 and CASPIAN trials showed that the addition of the anti-programmed death-ligand 1 (PD-L1) inhibitors atezolizumab and durvalumab to carboplatin–etoposide significantly improved both progression-free survival (PFS) and overall survival (OS), leading to the adoption of chemoimmunotherapy as the standard first-line treatment for ES-SCLC [7,8]. Although the use of immunotherapy alongside platinum etoposide in the first-line setting achieves initial tumor response rates of approximately 60–70%, the median OS for patients still remains approximately 12 to 13 months. [7,8].

The predictive role of PD-L1 expression, well established in NSCLC, is limited in SCLC due to its generally low expression levels, making it challenging to use as a reliable predictive or prognostic biomarker for immunotherapy response and efficacy [9,10]. Moreover, exploratory analyses from the IMpower133 trial revealed that PD-L1 expression is not a strong predictor of response to immune checkpoint inhibitors in SCLC [11].

Inflammatory biomarkers that may predict immunotherapy efficacy in ES-SCLC have been investigated in several previous studies. Elevated neutrophil-to-lymphocyte ratio (NLR), platelet-to-lymphocyte ratio (PLR), and systemic immune-inflammation index (SII) have generally been identified as indicators of poor prognosis [12,13]. Additionally, scoring systems such as the Lung Immune Prognostic Index (LIPI) and the Glasgow Prognostic Score (GPS) have been investigated and proposed as potentially significant prognostic indicators, with poor LIPI scores being associated with shorter PFS and OS, whereas favourable GPS scores correlated with better survival outcomes in patients with ES-SCLC receiving chemoimmunotherapy [14,15]. Nevertheless, a clinically validated and widely accepted predictive or prognostic biomarker for ES-SCLC has yet to be established. Therefore, in the present study, we sought to develop a novel prognostic classification by integrating LIPI and GPS in ES-SCLC patients treated with chemoimmunotherapy. We designated this composite model as the Combined Inflammatory Prognostic Score (CIPS) and aimed to evaluate its prognostic significance in this patient population.

## 2. Results

### 2.1. Patient Characteristics

The study included 166 patients, of whom 110 (66.3%) were male. The median age of patients in the study was 64 years (range, 30–90). A total of 156 patients (94%) had ES-SCLC at the time of initial diagnosis. The majority of patients had an ECOG PS of 0. (n = 115, 69.3%). Nearly all patients were either current or former smokers (n = 158, 95.2%). Metastatic involvement was most frequently observed in the bone (n = 94, 56.6%), distant lymph nodes (n = 62, 37.3%), and liver (n = 59, 35.5%). Baseline LIPI data were available for 123 patients. Among these, 46 (37.4%) were categorized as favourable LIPI, 55 (44.7%) as intermediate, and 22 (17.9%) as poor prognostic LIPI. Both GPS and CIPS scores were assessable in 120 patients. According to the GPS, 51 (42.5%) patients were classified as favourable, 59 (49.2%) as intermediate, and 10 (8.3%) as poor prognostic. Based on the Combined Inflammatory Prognostic Score (CIPS), 59 (49.2%) patients were assigned to the low-risk group, whereas 61 (50.8%) were categorized as high-risk. Baseline data for the study cohort are presented in Table 1.

Given that CRP and albumin may reflect both systemic inflammation and overall patient condition, their potential association with ECOG performance status was evaluated. No significant correlation was observed between ECOG performance status and CRP (ρ = 0.074, *p* = 0.343) or albumin (ρ = −0.079, *p* = 0.380), suggesting that these biomarkers may reflect biological processes independent of functional status.

### 2.2. Survival Outcomes and Treatment Responses

**Figure 1:**
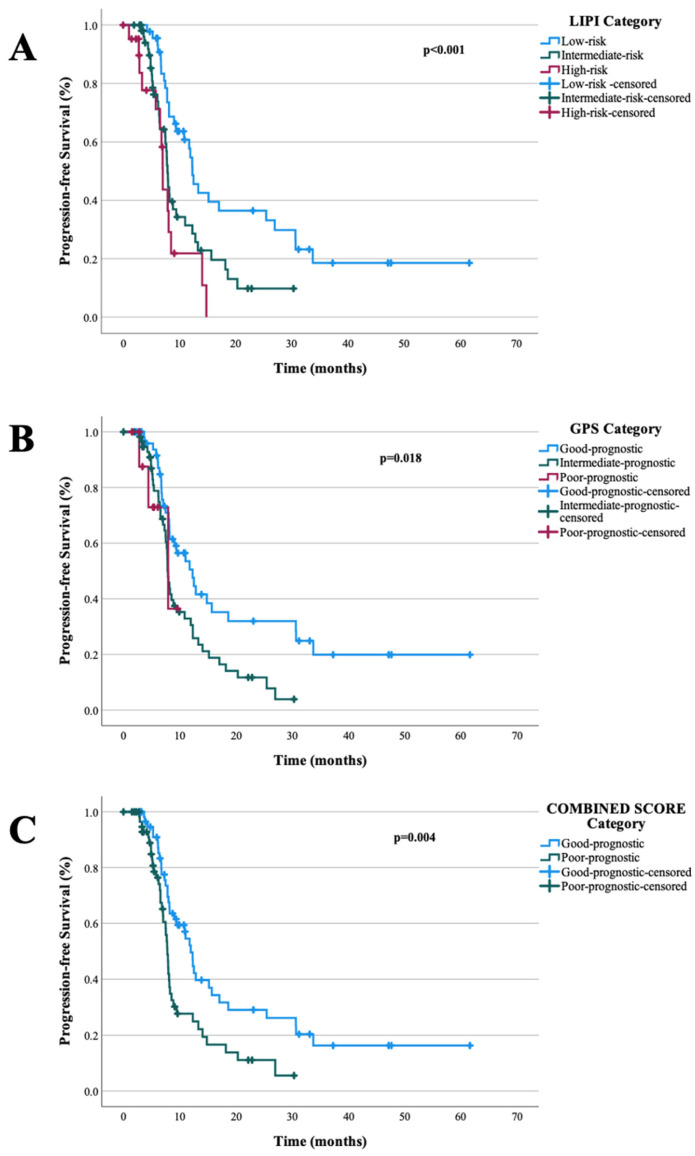
PFS curves according to the LIPI, GPS and CIPS categories; (**A**): LIPI, (**B**): GPS, (**C**): CIPS.

**Figure 2:**
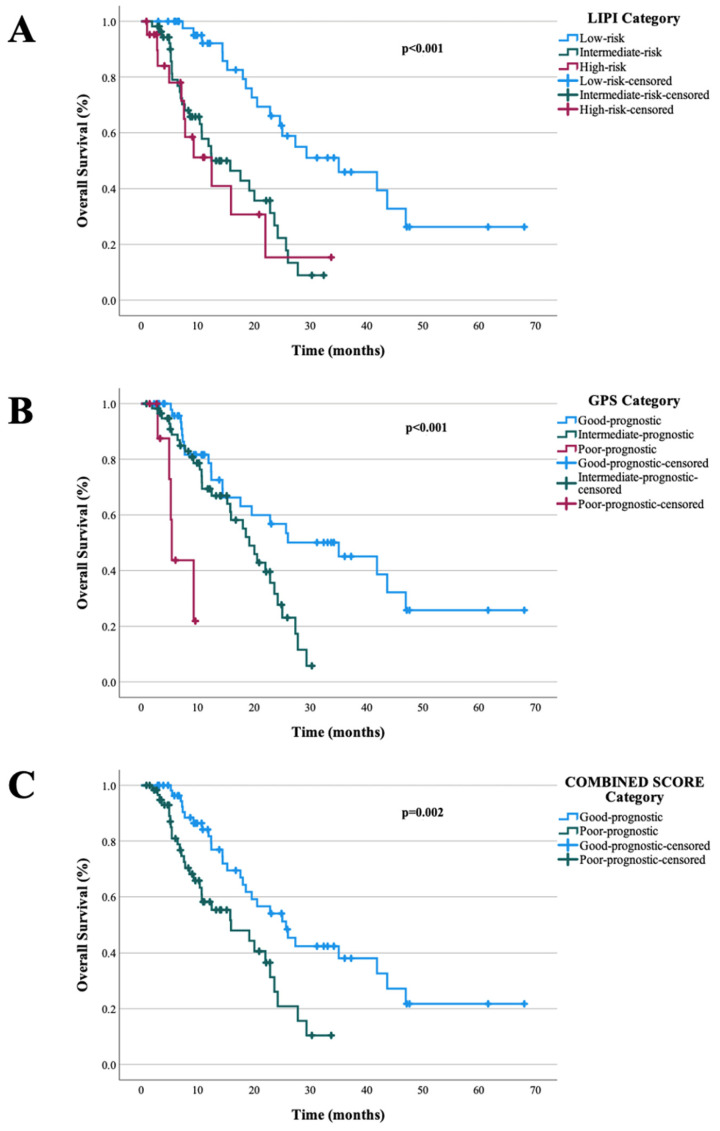
OS curves according to the LIPI, GPS and CIPS categories; (**A**): LIPI, (**B**): GPS, (**C**): CIPS.

The median follow-up duration for the cohort was 25.93 months. Median PFS in the cohort was 8.16 months (95% CI: 7.62–8.71). In the univariate analysis, poor ECOG PS (*p* = 0.022), the presence of bone metastasis (*p* = 0.003), and liver metastasis (*p* = 0.005) were identified as adverse prognostic factors for PFS. Furthermore, patients with a poor prognostic LIPI demonstrated significantly shorter PFS (*p* < 0.001), as did those with a poor prognostic GPS (*p* = 0.018). Similarly, classification into the high-risk CIPS group was associated with shorter PFS (*p* = 0.004). In the multivariate analysis for PFS, only poor prognostic LIPI (*p* = 0.023, HR, 1.739 CI 95% 1.081–2.797) and poor prognostic GPS (*p* = 0.041, HR, 1.764 CI 95% 1.024–3.04) remained as independent risk factors associated with shorter PFS. Univariable and multivariable analyses of factors associated with PFS summarized in Table 2. The median OS was 15.96 months (95% CI: 12.49–19.44). In the univariate analysis, poor ECOG PS (*p* = 0.005), presence of liver metastasis (*p* = 0.021), and presence of bone metastasis (*p* = 0.007) were identified as adverse prognostic factors for OS. Conversely, Patients with favourable LIPI (*p* < 0.001), favourable GPS (*p* < 0.001), and low-risk CIPS classification (*p* = 0.002) had significantly longer OS. In the multivariable analysis, among these factors, poor prognostic LIPI (*p* = 0.002; HR = 2.361; 95% CI: 1.367–4.078), poor prognostic GPS (*p* = 0.002; HR = 2.780; 95% CI: 1.460–5.313), and ECOG PS (*p* = 0.033; HR = 1.618; 95% CI: 1.039–2.518) remained significant predictors of shorter OS. LIPI, GPS, and CIPS scores showed prognostic value for PFS as well as OS in univariable analyses, whereas in multivariable models, only LIPI and GPS remained significant, while CIPS did not retain its prognostic impact. Table 3 demonstrates univariable and multivariable analyses of prognostic factors associated with OS. Figure 1 and Figure 2 illustrate the associations of LIPI, GPS, and CIPS classifications with PFS and OS, respectively.

**Figure 3:**
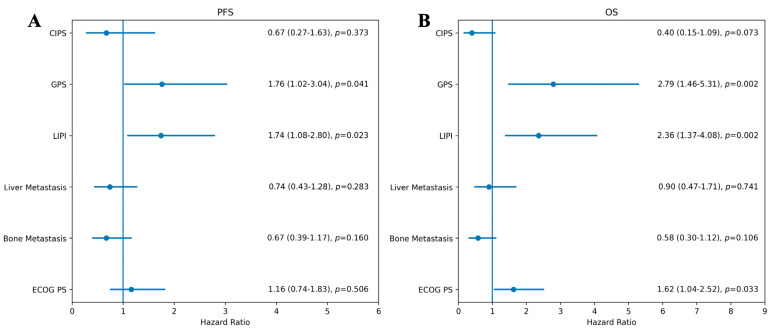
Forest plots of multivariable Cox regression analyses for PFS and OS. Panels (**A**,**B**) display PFS and OS, respectively. Hazard ratios (HRs) with 95% confidence intervals (CIs) are shown. A tabulated summary of HRs and *p*-values is provided alongside each plot.

Subgroup analyses according to the type of immunotherapy agent showed no significant heterogeneity in survival outcomes, although numerical differences were observed across groups. Given the limited sample size, these findings should be interpreted cautiously. The results of the multivariable Cox regression analyses for PFS and OS are presented as forest plots (Figure 3). Patients received a median of 4 chemotherapy cycles (range, 2–6) and 9 immunotherapy cycles (range, 2–58). Among the 166 patients, 16 (9.6%) achieved a CR, 125 (75.3%) achieved a PR, 5 (3.0%) had SD, and 20 (12.0%) experienced PD. The ORR was calculated as 84.9%, and the DCR as 88.0%.

## 3. Discussion

Findings from the IMpower133 and CASPIAN trials have led to the adoption of platinum-based chemotherapy combined with atezolizumab or durvalumab as the standard first-line approach for ES-SCLC [7,8]. Moreover, the longer follow-up update of the IMpower133 trial demonstrated a median PFS of 5.2 months (95% CI, 4.4–5.6) and a median OS of 12.3 months (95% CI, 10.8–15.8) [11]. In our cohort, median PFS was 8.16 months (95% CI: 7.62–8.71), while median OS was 15.96 months (95% CI: 12.49–19.44). These survival outcomes were longer than those reported in previously published real- world data from Turkey, in which the median PFS and OS were 6.8 months (95% CI: 5.7–7.8) and 11.9 months (95% CI: 11–12.7), respectively [16]. The longer PFS observed in our cohort may be attributed to a relatively extended follow-up period. The higher OS, on the other hand, may be explained by the use of effective second-line treatment options such as tarlatamab and lurbinectedin, as well as by the successful management of oligoprogressive disease during maintenance therapy through local treatment modalities [17,18]. However, it should be noted that these local interventions were not included as covariates in the survival analyses or multivariate models, and therefore their potential impact on OS was not formally adjusted for.

Zhou et al. showed that patients with poorer ECOG PS had worse survival outcomes in terms of both PFS and OS [19]. Similarly, in our study, patients with poorer ECOG PS exhibited significantly shorter PFS and OS. Although this parameter did not maintain statistical significance for PFS in the multivariable analysis, it remained an independent prognostic factor for OS (*p* = 0.033; HR = 1.618; 95% CI: 1.039–2.518). The presence of liver metastasis has previously been associated with shorter PFS and OS, and a comparable finding was observed in our cohort; nonetheless, in the multivariable analysis, liver metastasis did not retain its significance as an independent prognostic factor [20]. Previous data have demonstrated that patients with bone metastases have shorter OS compared to those without bone involvement [21]. Consistent with the literature, our study also showed that patients with bone metastases had shorter PFS and OS than in those without. However, in the multivariable analysis, the presence of bone metastasis did not remain an independent prognostic factor. Metastatic sites other than the liver and bone, including the brain, distant lymph nodes, and adrenal glands, were not associated with PFS or OS. Consistent with previous data, patients with bone and liver metastases had shorter median OS compared with those who had brain metastases (12.53 vs. 18.06 months, respectively) [22]. In our study, the absence of independent prognostic significance for liver and bone metastases in the multivariable analysis may be attributed to the fact that patients with these metastatic sites tended to have poorer ECOG PS and more unfavorable LIPI and GPS profiles, and these variables were already included in the multivariable model.

Recent studies have consistently demonstrated that a higher LIPI score is associated with significantly shorter PFS and OS [14,23]. Concordantly, in our study, patients with a favourable LIPI score exhibited markedly longer PFS and OS. In the multivariate analysis, LIPI retained its prognostic significance and emerged as an independent predictor of both PFS (*p* = 0.023) and OS (*p* = 0.002). Furthermore, the HR for PFS was similar to that reported in the literature (HR 1.739, 95% CI: 1.081–2.797 vs. HR 1.57, 95% CI: 1.20–2.06), whereas the HR for OS was higher in our study compared with previous reports (HR 2.361, 95% CI: 1.367–4.078 vs. HR 1.76, 95% CI: 1.26–2.45) [14]. In several recent studies, the prognostic significance of the GPS has been evaluated, and higher scores have been associated with shorter PFS and OS [15,24]. Consistent with these findings, our study demonstrated that patients with favourable GPS scores had longer PFS and OS durations. In the multivariate analysis, the GPS remained an independent prognostic factor for both PFS (*p* = 0.041) and OS (*p* = 0.002). Moreover, while the HR for PFS was comparable to that reported in the literature (HR 1.764, 95% CI: 1.024–3.04 vs. HR 1.83, 95% CI: 1.39–2.42), the HR for OS was notably higher in our cohort compared with previous reports (HR 2.780, 95% CI: 1.460–5.313 vs. HR 1.83, 95% CI: 1.39–2.42) [25].

From a clinical perspective, LIPI and GPS are well-established inflammatory indices that provide meaningful prognostic information in patients with ES-SCLC receiving chemoimmunotherapy. In routine practice, these scores may assist clinicians in identifying patients who require closer follow-up, more intensive monitoring, and earlier consideration of second line treatment strategies or clinical trial enrollment. However, despite these advantages, their clinical applicability may be limited by heterogeneous patient distribution across risk categories and the potential overlap of survival outcomes within intermediate-risk groups, which may reduce their practicality in individual-level decision-making. In addition to LIPI and GPS, several composite indices integrating inflammatory, nutritional, and clinical parameters, such as the CALLY index (C-reactive protein–albumin–lymphocyte index) and the Advanced Lung Cancer Inflammation Index (ALI; calculated as BMI × albumin/NLR), have also been proposed in the literature. The CALLY index has been evaluated in patients with NSCLC and has been shown to serve as a significant prognostic marker for OS [26]. Similarly, ALI has been investigated in SCLC and has been reported to demonstrate a stronger association with survival outcomes compared with CALLY [27]. However, it is important to note that in both of these studies, the patient populations consisted of individuals who did not receive chemoimmunotherapy, which currently represents the standard first-line treatment [26,27].

In this context, the CIPS model was developed by integrating LIPI and GPS to provide a more simplified and potentially more practical approach to risk stratification. The rationale for this approach lies in the complementary biological roles of the included parameters. By combining tumor-related inflammatory markers (dNLR and LDH) with host-related inflammatory and nutritional indicators (CRP and albumin), CIPS reflects both tumor burden and the systemic host response. In this way, it captures multiple dimensions of cancer-related inflammation within a single framework, offering a more comprehensive assessment compared with individual biomarkers alone.

The distribution of patients according to the total number of adverse factors (derived from LIPI (dNLR > 3 and LDH above the ULN) and GPS (elevated CRP and hypoalbuminemia) was as follows: 23 patients had 0 factors, 38 had 1 factor, 42 had 2 factors, 15 had 3 factors, and 2 had 4 factors. This distribution demonstrated clustering within a limited range and a relatively small number of patients in the highest-risk categories. The contribution of individual components varied across patients, reflecting the multifactorial structure of the CIPS score. Based on this distribution and to avoid sparsely populated subgroups, a two tier classification (0–1 vs. 2–4) was considered the most appropriate approach. This approach was also supported by a clinically intuitive rationale, whereby patients classified as intermediate or high risk according to either LIPI or GPS were more likely to have an unfavorable prognosis. Accordingly, combining these scores into a binary structure allowed identification of patients with an overall higher inflammatory burden, even when only one of the component scores indicated increased risk. Exploratory analyses using alternative cut-off strategies demonstrated overlapping survival patterns among intermediate groups, further supporting the use of a simplified two-tier classification.

CIPS demonstrated a clear ability to stratify patients into two distinct risk groups with significantly different survival outcomes in univariable and Kaplan–Meier analyses. Patients classified in the low-risk group exhibited significantly longer PFS (11.96 vs. 7.8 months) and OS (25.73 vs. 11.96 months) compared with those in the high-risk group, with an early and sustained separation of survival curves throughout follow-up. However, CIPS did not retain prognostic significance in multivariable analysis. This finding may be explained not only by the relatively limited sample size but also by the inherent overlap and collinearity between the composite score and its individual components included in the model. Given that CIPS is derived from LIPI and GPS, which were also incorporated into the multivariable analysis, this result is not unexpected. Despite this, the consistent separation observed in survival analyses suggests that CIPS still captures clinically meaningful prognostic information. Compared with LIPI or GPS alone, combining these indices into a binary structure may reduce heterogeneity within intermediate-risk groups and allow for a more distinct separation of clinically meaningful risk categories, thereby enhancing practical prognostic discrimination in routine clinical practice. From a practical perspective, the simplified two-tier structure and more balanced patient distribution of CIPS may further enhance its usability in routine clinical settings. In particular, it may facilitate rapid risk stratification, support identification of patients who may benefit from closer monitoring or earlier therapeutic adjustments, and assist clinicians in discussing prognosis with patients and their families. In this way, CIPS may contribute to more individualized treatment planning and a more patient-centered approach to care in ES-SCLC.

To the best of our knowledge, aside from our study, there are no published data in the literature evaluating patients with ES-SCLC treated with first-line platinum-based chemotherapy plus immunotherapy in which the prognostic impact of LIPI and GPS has been jointly assessed. Our study not only demonstrated the prognostic relevance of both scores individually but also uniquely integrated their key components, dNLR, LDH, CRP, and albumin into a combined model. This comprehensive evaluation highlights the novelty of our approach and provides a distinct contribution to the current understanding of prognostic assessment in ES-SCLC.

Several limitations of this study should be considered. First, the retrospective design is inherently susceptible to bias and unmeasured confounding, which may have influenced the interpretation of the findings. However, to reduce the impact of potential selection bias, all consecutive patients who met the predefined inclusion criteria were included. In addition, baseline characteristics (including age, sex, ECOG PS, and metastatic sites) and survival outcomes were compared between patients included in the biomarker analyses and those excluded due to missing data, and no significant differences were observed, suggesting that selection bias related to missing data was limited. Missingness was considered likely to be non-random due to the retrospective nature of data collection, which may further limit generalizability. Second, the study was conducted in only two centers, which may limit the broader applicability of the findings. Third, the relatively small patient population represents another limitation. Fourth, the use of two different immunotherapy agents and the predominance of atezolizumab in the cohort may have introduced a degree of heterogeneity. However, no significant difference in survival outcomes was observed between patients treated with atezolizumab and those receiving durvalumab (PFS, *p* = 0.720; OS, *p* = 0.240). Fourth, in our study, LIPI and GPS were calculated using predefined thresholds established in prior literature rather than cohort-specific cutoffs. Although data-driven approaches such as ROC analysis may improve discrimination within a given dataset, they may also introduce overfitting and limit generalizability, particularly in retrospective cohorts with limited sample size. Therefore, we prioritized the use of standardized thresholds to ensure comparability with previous studies and to enhance the clinical applicability of our findings. Future prospective studies may further investigate whether optimized cutoffs provide incremental prognostic value. The retrospective design of this study inherently introduces the risk of selection bias and limits causal interpretation. In addition, missing data for key inflammatory indices, including LIPI and GPS/CIPS, reduced the effective sample size in subgroup analyses. Although sensitivity analyses suggested consistent results, the possibility of residual bias cannot be excluded. On the other hand, the lack of formal discrimination analysis (e.g., C-index) was another limitation of this study and should be addressed in future studies with larger cohorts. Therefore, our findings should be interpreted with caution and require validation in larger, prospective cohorts. Finally, we did not perform decision curve analysis, as our study was not designed to develop a clinical prediction model. Future studies with externally validated models should assess clinical utility using DCA.

## 4. Materials and Methods

### 4.1. Study Design and Patient Selection

**Figure 4:**
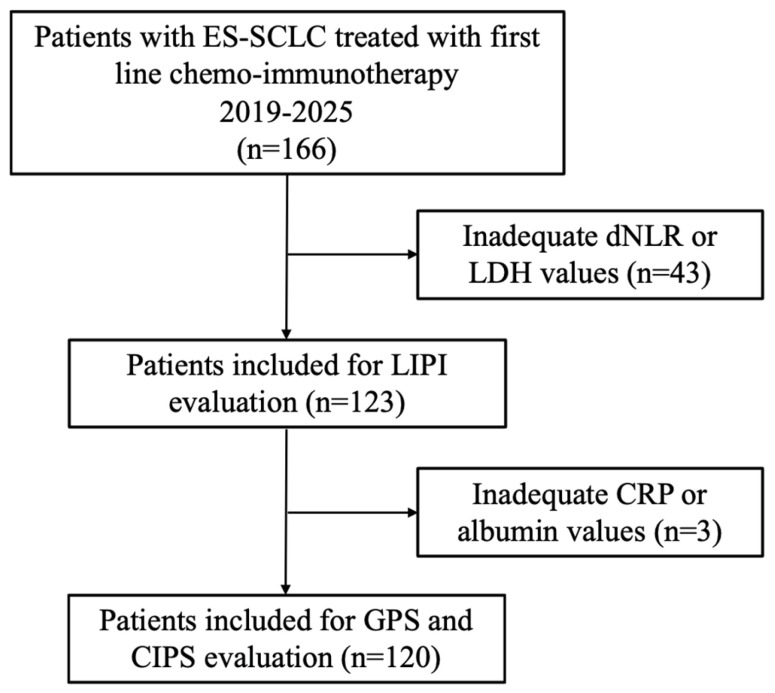
Patient selection and biomarker assessment flowchart. Abbreviations: ES-SCLC, Extensive stage Small-Cell Lung Cancer; dNLR, Derived Neutrophil-to-Lymhocyte Ratio; LIPI, Lung Immune Prognostic Index; GPS, Glasgow Prognostic Score; CIPS, Combined Inflammatory Prognostic Score.

Clinical data of patients aged ≥18 years with a diagnosis of ES-SCLC who were treated in the first-line setting with platinum–etoposide in combination with either atezolizumab or durvalumab between 2019 and 2025 at the Medical Oncology Departments of Istanbul Medipol University and Koç University were retrospectively analyzed. Patients treated with immunotherapy in the second line setting or in combination with irinotecan were excluded from the study. Patient staging was conducted according to the 8th edition of the American Joint Committee on Cancer (AJCC) and the Union for International Cancer Control (UICC) staging system, based on an integrated assessment of clinical and radiological findings. A total of 166 patients who met the following criteria were included in the study: age over 18 years, diagnosis of ES-SCLC, receipt of first-line chemoimmunotherapy and an Eastern Cooperative Oncology Group performance status (ECOG PS) of 0–2 [28]. Among the 166 patients included in the study, dNLR (derived neutrophil-to-lymphocyte ratio) and LDH (lactate dehydrogenase) values required for calculating the LIPI score were available in 123 patients. CRP (C-reactive protein) and albumin levels, which are necessary for computing the GPS, were available in 120 patients; therefore, GPS and CIPS scores were calculated in these 120 patients. Figure 4 shows the flow chart of patient selection.

### 4.2. Treatment Protocols

Patients received first-line treatment with either carboplatin (area under the curve of 5 mg/mL/min) or cisplatin (75 mg/m^2^), administered intravenously on day 1, in combination with etoposide (100 mg/m^2^, intravenously on days 1–3) and either atezolizumab (1200 mg intravenously on day 1) or durvalumab (1500 mg intravenously on day 1), in 3-week cycles. Patients without disease progression following 4–6 cycles of combination therapy proceeded to maintenance treatment with atezolizumab (1200 mg intravenously every 3 weeks) or durvalumab (1500 mg intravenously every 4 weeks), which was continued until disease progression, unacceptable toxicity, or death.

### 4.3. Data Collection

The following patient data were collected from clinical records: sex, age at diagnosis, smoking history, disease stage at diagnosis, ECOG PS, metastatic sites, administered chemotherapy regimen (cisplatin + etoposide or carboplatin + etoposide), the type of immunotherapy agent (atezolizumab or durvalumab), and baseline laboratory parameters, including dNLR, LDH, albumin, and CRP levels, obtained within one week prior to treatment initiation. LIPI and GPS scores were subsequently calculated using these parameters. Biomarker assessments were performed in the absence of clinically evident infection or acute inflammatory conditions. Patients with documented use of systemic corticosteroids or antibiotics within one week prior to biomarker measurement were not included. In patients with transient inflammatory processes, laboratory values obtained after clinical resolution were used.

### 4.4. Efficacy

The primary efficacy endpoints of the study were objective response rate (ORR), PFS and OS. PFS was defined as the duration from treatment onset to the first documented disease progression or death from any cause, with patients censored at their last known follow-up if no event occurred. OS was measured from the start of treatment to death from any cause; patients who were alive at the time of analysis were censored at their last known follow-up. Tumor response was assessed according to the Response Evaluation Criteria in Solid Tumors (RECIST), version 1.1 [29]. Treatment outcomes were classified as complete response (CR), partial response (PR), stable disease (SD), or progressive disease (PD). The objective response rate (ORR) represented the proportion of patients achieving either a CR or PR, whereas the disease control rate (DCR) included all patients who achieved CR, PR, or maintained SD as their best overall response [29].

### 4.5. Predictive and Prognostic Markers

Missing data were present for inflammatory indices, including LIPI and GPS/CIPS. Analyses involving these variables were therefore conducted on available cases (complete-case approach). To evaluate the potential impact of missing data, sensitivity analyses were performed by comparing baseline characteristics and survival outcomes between patients with complete data and the overall study population. LIPI was derived from two parameters: dNLR and serum LDH level. dNLR greater than 3 and/or an LDH level above the upper limit of normal (ULN) were considered adverse prognostic factors. Patients were stratified into three groups according to the number of adverse factors: good LIPI (0 factors), intermediate LIPI (1 factor), and poor LIPI (2 factors). GPS was determined using baseline serum levels of albumin and CRP. Patients with CRP ≤10 mg/L and albumin ≥3.5 g/dL were assigned a GPS of 0. Those with either CRP >10 mg/L or Albumin <3.5 g/dL were assigned a GPS of 1, while patients with both elevated CRP and low albumin were given a GPS of 2. Accordingly, we combined the LIPI and GPS scores to establish the Combined Inflammatory Prognostic Score (CIPS). This scoring system incorporated four baseline parameters: dNLR > 3, LDH > ULN, CRP > 10 mg/L, and albumin < 3.5 g/dL. Patients with none or only one of these adverse factors were classified as having low-risk CIPS, whereas those with two or more positive parameters were categorized as having high-risk CIPS. The definitions and risk stratification criteria for LIPI, GPS, and the newly developed CIPS are explained in Table 4.

### 4.6. Statistical Analysis

All data obtained from participating centers were pooled for statistical evaluation. All data obtained from participating centers were pooled for statistical evaluation. Study characteristics were described using appropriate summary measures, including counts and percentages for categorical variables, and mean with standard deviation or median with range for continuous variables, depending on distribution. To evaluate potential overlap between clinical performance status and biomarkers, Spearman correlation analysis was performed between ECOG PS and CRP/albumin levels.

Time-to-event outcomes were assessed using Kaplan–Meier survival estimates, with comparisons across groups performed using the log-rank test. The association between clinical parameters and survival endpoints (PFS and OS) was examined using Cox proportional hazards models. Missing data were handled using a complete-case approach. Patients with missing values in variables included in the regression models were excluded from the relevant analyses, as the retrospective design and the limited sample size did not allow for robust multiple imputation. For survival analyses, univariable Cox proportional hazards regression was initially performed for candidate variables. Variables considered clinically relevant and/or showing an association in univariable analysis were entered into the multivariable model using a stepwise selection procedure. The proportional hazards assumption was assessed using schoenfeld residuals and graphical examination of log-minus-log survival plots. No substantial violation of the proportional hazards assumption was observed. Variables with a *p*-value < 0.10 in univariate Cox regression analysis were considered for inclusion in the multivariate model. Additionally, clinically relevant variables were included regardless of statistical significance. To avoid overfitting, the number of covariates was limited according to the number of events.

Determinants of treatment response were analyzed using binary logistic regression. Effect sizes were presented as hazard ratios (HRs) along with 95% confidence intervals (CIs). The proportional hazards assumption was assessed using graphical methods (log-minus-log plots) and Schoenfeld residuals. No significant violations were detected. Statistical analyses were performed using IBM SPSS Statistics (version 27.0; IBM Corp., Armonk, NY, USA), and statistical significance was defined as a two-sided *p* value < 0.05.

## 5. Conclusions

In summary, the present study identified the LIPI and GPS scores as independent prognostic indicators for PFS, while ECOG PS, in addition to these parameters, emerged as an independent prognostic factor for OS. Although CIPS did not maintain statistical significance in multivariate analysis, its clear prognostic separation, ease of calculation, and integration of four routinely available biomarkers indicate its potential for clinical use pending external validation. To the best of our knowledge, this study is the first comprehensive analysis to evaluate such an integrated prognostic scoring approach in ES-SCLC patients treated with chemoimmunotherapy. Further large-scale, multicenter, and long-term prospective studies are warranted to validate these findings and to explore the potential incorporation of the CIPS score into routine clinical practice.

## Figures and Tables

**Table 1:** Baseline Characteristics of the Study Cohort.

Characteristic	n (%)
Age	Median: 64 (range, 30–90)
Sex	
Male	110 (66.3)
Female	56 (33.7)
ECOG PS	
0	115 (69.3)
1	43 (25.9)
2	8 (4.8)
Smoking history	
Never smoked	8 (4.8)
Ex-smoker	84 (50.6)
Current smoker	74 (44.6)
Disease status at first diagnosis	
ES-SCLC	156 (94)
LS-SCLC	10 (6)
Site of metastasis	
Bone	94 (56.6)
Distant lymph nodes	62 (37.3)
Liver	59 (35.5)
Brain	46 (27.7)
Adrenal	29 (17.5)
Chemotherapy regimen	
Carboplatin + Etoposide	137 (82.5)
Cisplatin + Etoposide	29 (17.5)
Immunotherapy agent	
Atezolizumab	140 (84.3)
Durvalumab	26 (15.7)
LIPI	n = 123
Favourable	46 (37.4)
Intermediate	55 (44.7)
Poor prognostic	22 (17.9)
GPS	n = 120
Favourable	51 (42.5)
Intermediate	59 (49.2)
Poor prognostic	10 (8.3)
CIPS	n = 120
Low risk	59 (49.2)
High risk	61 (50.8)

Abbreviations: ECOG, Eastern Cooperative Oncology Group; ES-SCLC, Extensive stage Small-Cell Lung Cancer; LS-SCLC, Limited Stage Small-Cell Lung Cancer; LIPI, Lung Immune Prognostic Index; GPS, Glasgow Prognostic Score; CIPS, Combined Inflammatory Prognostic Score.

**Table 2:** Univariable and multivariable analysis for PFS.

Features	n (%)	Median PFS (Months)	Univariate *p*	Multivariate *p*	HR (CI%)
Sex			0.330		
Male	110 (66.3)	8.16			
Female	56 (33.7)	7.96			
ECOG PS			0.020	0.506	1.16 (0.74–1.83)
0	115 (69.3)	8.1			
1	43 (25.9)	9.06			
2	8 (4.8)	6.23			
Smoking history			0.077		
Current smoker	74 (44.6)	5.23			
Ex-smoker	84 (50.6)	8.46			
Never smoked	8 (4.8)	8			
Disease status at first diagnosis			0.263		
ES-SCLC	156 (94)	8.16			
LS-SCLC	10 (6)	7.36			
Bone Metastasis			0.003	0.160	0.67 (0.39–1.17)
Present	94 (56.6)	7.83			
Absent	72 (43.4)	10.86			
Liver Metastasis			0.005	0.283	0.74 (0.43–1.28)
Present	59 (35.5)	7.76			
Absent	107 (64.5)	8.53			
Site of metastasis					
Distant lymph nodes	62 (37.3)	8.2	0.650		
Brain	46 (27.7)	7.9	0.670		
Adrenal	29 (17.5)	7.76	0.470		
Chemotherapy regimen			0.790		
Carboplatin + etoposide	137 (82.5)	8.1			
Cysplatin + etoposide	29 (17.5)	8.53			
Immunotherapy agent			0.720		
Atezolizumab	140 (84.3)	8.03			
Durvalumab	26 (15.7)	8.86			
LIPI Class	n = 123		<0.001	0.023	1.74 (1.08–2.80)
Favourable	46 (37.4)	12.33			
Intermediate	55 (44.7)	7.9			
Poor prognostic	22 (17.9)	7.06			
GPS Class	n = 120		0.018	0.041	1.76 (1.02–3.04)
Favourable	51 (42.5)	12.3			
Intermediate	59 (49.2)	7.83			
Poor prognostic	10 (8.3)	7.93			
CIPS Score	n = 120		0.004	0.373	0.67 (0.27–1.63)
Low risk	59 (49.2)	11.96			
High risk	61 (50.8)	7.8			

Abbreviations: ECOG, Eastern Cooperative Oncology Group; ES-SCLC, Extensive stage Small-Cell Lung Cancer; LS-SCLC, Limited Stage Small-Cell Lung Cancer; LIPI, Lung Immune Prognostic Index; GPS, Glasgow Prognostic Score; CIPS, Combined Inflammatory Prognostic Score.

**Table 3:** Univariable and multivariable analysis for OS.

Features	n (%)	Median OS (Months)	Univariate *p* Value	Multivariate *p* Value	HR (CI%)
Sex			0.904		
Male	110 (66.3)	18.43			
Female	56 (33.7)	14.43			
ECOG PS			0.005	0.033	1.62 (1.04–2.52)
0	115 (69.3)	18.66			
1	43 (25.9)	12.53			
2	8 (4.8)	7.8			
Smoking history			0.208		
Current smoker	74 (44.6)	15.83			
Ex-smoker	84 (50.6)	18.66			
Never smoked	8 (4.8)	9.63			
Disease status at first diagnosis			0.684		
ES-SCLC	156 (94)	15.96			
LS-SCLC	10 (6)	21.93			
Bone Metastasis			0.007	0.106	0.58 (0.30–1.12)
Present	94 (56.6)	12.53			
Absent	72 (43.4)	23.66			
Liver Metastasis			0.021	0.741	0.90 (0.47–1.71)
Present	59 (35.5)	12.53			
Absent	107 (64.5)	19.56			
Site of metastasis					
Distant lymph nodes	62 (37.3)	19.56	0.939		
Brain	46 (27.7)	18.06	0.324		
Adrenal	29 (17.5)	17.96	0.956		
Chemotherapy regimen			0.194		
Carboplatin + etoposide	137 (82.5)	15.23			
Cysplatin + etoposide	29 (17.5)	22.06			
Immunotherapy agent			0.240		
Atezolizumab	140 (84.3)	15.3			
Durvalumab	26 (15.7)	NR			
LIPI Class	n = 123		<0.001	0.002	2.36 (1.37–4.08)
Favourable	46 (37.4)	35.1			
Intermediate	55 (44.7)	12.46			
Poor prognostic	22 (17.9)	12.53			
GPS Class	n = 120		<0.001	0.002	2.79 (1.46–5.31)
Favourable	51 (42.5)	35.1			
Intermediate	59 (49.2)	19.2			
Poor prognostic	10 (8.3)	5.43			
CIPS Score	n = 120		0.002	0.073	0.40 (0.15–1.09)
Low risk	59 (49.2)	25.73			
High risk	61 (50.8)	15.96			

Abbreviations: ECOG, Eastern Cooperative Oncology Group; ES-SCLC, Extensive stage Small-Cell Lung Cancer; LS-SCLC, Limited Stage Small-Cell Lung Cancer; LIPI, Lung Immune Prognostic Index; GPS, Glasgow Prognostic Score; CIPS, Combined Inflammatory Prognostic Score.

**Table 4:** Definitions and Risk Stratification of LIPI, GPS, and CIPS.

Scoring System	Risk Category	Definition/Criteria
LIPI	Favourable	dNLR ≤ 3 and LDH ≤ ULN
	Intermediate	Either dNLR > 3 or LDH > ULN
	Poor	dNLR > 3 and LDH > ULN
GPS	Favourable	CRP ≤ 10 mg/L and Albumin ≥ 3.5 g/dL
	Intermediate	Either CRP > 10 mg/L or Albumin < 3.5 g/dL
	Poor	CRP > 10 mg/L and Albumin < 3.5 g/dL
CIPS	Low Risk	0–1 of the following: dNLR > 3, LDH > ULN, CRP > 10 mg/L, Albumin < 3.5 g/dL
	High Risk	2–4 of the above risk factors present

Abbreviations: CIPS, Combined Inflammatory Prognostic Score; CRP, C-Reactive Protein; dNLR, Derived Neutrophil-to-Lymphocyte Ratio; GPS, Glasgow Prognostic Score; LDH, Lactate Dehydrogenase; LIPI, Lung Immune Prognostic Index; ULN, Upper Limit of Normal.

## Data Availability

The data presented in this study are available from the corresponding author upon request. Due to the sensitive nature of the data, they are not publicly accessible.
